# Lack of Functional Benefit with Glutamine versus Placebo in Duchenne Muscular Dystrophy: A Randomized Crossover Trial

**DOI:** 10.1371/journal.pone.0005448

**Published:** 2009-05-06

**Authors:** Elise Mok, Guy Letellier, Jean-Marie Cuisset, André Denjean, Frédéric Gottrand, Corinne Alberti, Régis Hankard

**Affiliations:** 1 INSERM Centre D'Investigation Clinique 802, CHU de Poitiers, Poitiers, France; 2 Pédiatrie Multidisciplinaire – Nutrition de l'Enfant, CHU de Poitiers, Poitiers, France; 3 INSERM Centre D'Investigation Clinique 9202, Assistance Publique-Hôpitaux de Paris, Hôpital Robert Debré, Paris, France; 4 INSERM Centre D'Investigation Clinique 9301, CHR&U de Lille, Lille, France; 5 EA3813 Laboratoire Adaptation Physiologique aux Activités Physiques, Université de Poitiers, Poitiers, France; 6 Unité d'Epidémiologie Clinique et INSERM, CIE5, Assistance Publique-Hôpitaux de Paris, Hôpital Robert Debré, Paris, France; 7 Service de Neuropédiatrie et Centre de Référence National des Maladies Neuromusculaires, CHR&U de Lille, Hôpital Roger-Salengro, Lille, France; 8 EA 3925, IFR 114, Faculté de Médecine, Université Lille 2, Lille, France; Baylor College of Medicine, United States of America

## Abstract

**Background:**

Oral glutamine decreases whole body protein breakdown in Duchenne muscular dystrophy (DMD). We evaluated the functional benefit of 4 months oral glutamine in DMD.

**Methodology/Principal Findings:**

30 ambulant DMD boys were included in this double-blind, randomized crossover trial with 2 intervention periods: glutamine (0.5 g/kg/d) and placebo, 4 months each, separated by a 1-month wash-out, at 3 outpatient clinical investigation centers in France. Functional benefit was tested by comparing glutamine versus placebo on change in walking speed at 4 months. Secondary outcome measures were: 2-minute walk test, work, power, muscle mass (urinary creatinine), markers of myofibrillar protein breakdown (urinary 3-methyl-histidine/creatinine), serum creatine phospho-kinase, body composition (fat free mass, fat mass percentage), safety and oral nutrient intake. There was no improvement in the primary end point (walking speed) or in secondary measures of muscle function (2-minute walk test, work, power) in the glutamine group compared with placebo. However, subjects receiving glutamine or placebo showed no deterioration in functional measures over the course of the 9-month trial. No differences in muscle mass, markers of protein breakdown or serum creatine phosho-kinase were observed, except for a blunted increase in fat free mass in the glutamine group which led to a greater increase in fat mass percentage. Glutamine was safe and well-tolerated.

**Conclusions:**

This trial did not identify additional benefit of 4 months oral glutamine over placebo on muscle mass or function in ambulatory DMD boys. Although apparently safe, current data cannot support routine supplementation in this population as a whole, until further research proves otherwise.

**Trial Registration:**

ClinicalTrials.gov NCT00296621

## Introduction

Duchenne muscular dystrophy (DMD) is a serious X-linked disease caused by mutations in the gene that encodes the cytoskeletal protein dystrophin [1]. The absence of dystrophin is associated with a progressive and severe loss of muscle mass and function. To date, there is no cure, emphasizing the need for the development of therapies that target downstream events in the pathologic progression of DMD muscle wasting. Although corticosteroids have been proven effective in preserving muscle mass and function in DMD [2], some physicians and families are reluctant to place children on this therapy owing to adverse effects. Nutritional therapies in conjunction with drug, cellular or gene therapy might provide another approach [3], [4]. However much of the data showing benefits are based on experimental evidence in the *mdx* mouse model [3]–[15], and few randomized controlled trials (RCT) have been conducted in DMD patients [16]–[19].

Glutamine (Gln) is a non-essential amino acid that is mainly produced and stored in skeletal muscle [20]. Evidence for its efficacy is supported by clinical benefits in several catabolic states [21]. Whole body Gln turnover is decreased in DMD compared to control children, resulting from a decrease in estimates of Gln *de novo* synthesis [22], which might reflect a decrease in muscle Gln production. Also because in protein-wasting situations (e.g. DMD) the intramuscular Gln concentration is low [23], Gln might be considered a conditionally essential amino acid. Acute oral Gln administered continuously for 5 hours decreased whole body protein breakdown in DMD boys [24]. This protein-sparing effect persisted when Gln was administered daily using the same dose (0.5 g/kg/d) over a longer period (10 days) [19]. Based on this evidence, the hypothesis was that supplemental Gln would slow disease progression and therefore provide functional benefit. To evaluate functional benefit, we compared change in walking speed after 4 months Gln versus placebo in ambulant DMD boys. Secondary endpoints were: 2-minute walk test (2-MWT), work, power, muscle mass, markers of myofibrillar protein breakdown, serum creatine phospho-kinase (CPK) and body composition. Safety and tolerance were also assessed.

## Methods

The protocol for this trial and supporting CONSORT checklist are available as supporting information; see Protocol S1 and Checklist S1.

### Participants

This was a randomized, placebo-controlled, double-blind, crossover trial of oral Gln versus placebo in ambulant DMD boys in 3 participating clinical investigation centers across France. The protocol was approved by the Paris-Bichat Ethics Committee (Comité Consultatif pour la Protection de la Personne dans la Recherche Biomédicale) and conducted according to Declaration of Helsinki principles. The statement from the Ethics Committee indicating their approval of the research is available as supporting information; see Statement S1. Subjects were selected on the basis of the following eligibility criteria: Pediatric neurologist diagnosis of DMD by clinical history, muscle biopsy (absence of dystrophin confirmed by immunohistochemistry or Western blot) and molecular biology, as well as the ability to walk ≥170 m. Exclusion criteria included: wheelchair dependency, body weight >60 kg, hepatic or renal insufficiency, or surgery scheduled in the year following the first visit. Subjects were recruited among those followed in multidisciplinary outpatient facilities from University hospitals in Paris, Poitiers and Lille, France. All participating families gave their written informed consent after a thorough explanation of the study protocol to both the parents and the children. A sample of a patient consent form (parent) and a patient assent form (children) are available as supporting information; see Form S1 and Form S2.

### Randomization and Interventions

Randomization and blinding were coordinated through the Département de la Recherche Clinique et du Développement of the Assistance Publique-Hôpitaux de Paris and a central pharmacy (Agence Générale des Equipements et Produits de Santé; AGEPS; Paris, France) who did not participate in the evaluation of study subjects. This included computerized generation of the allocation schedule in blocks of 6 subjects and blinded disbursement of treatment in numbered containers. The code was located at the Département de la Recherche Clinique et du Développement during the trial and until the completion of statistical analysis. No adverse events during the trial required breaking the code. Individual subjects were enrolled by the site investigator and were randomly assigned in a double blind crossover manner to 4 months of taking oral Gln (0.5 g/kg/d) followed by 4 months of taking placebo (maltodextrin; of equal weight) or vice versa. The two 4-month periods were separated by a 1-month wash-out. The order of treatment allocation was randomized. Participants, investigators, physicians, assessors and data analysts were blinded throughout the trial to the order of treatment assignment. The Gln dose (0.5 g/kg/d) was selected based on previous studies in DMD boys [24] and in healthy humans [25], [26] that showed a 2-fold increase in plasma Gln concentrations during Gln administration. This same dose decreased whole body protein degradation in DMD boys during Gln administration [24], which persisted 24 hours after supplementation ceased, while plasma Gln concentrations had returned to normal [19].

L-Glutamine and placebo were prepared by a central pharmacy (AGEPS) and kept by the pharmacist, who was not familiar with the subjects. The 2 supplements were prepared as powders of identical taste, odor and appearance and were flavored with banana, caramel and artificial sweetener (Aspartame; AGEPS). Supplements were identically packaged in 5 g sachets and disbursed to participants in 2 individual boxes (1 box representing a 1-month supply of Gln or placebo) which contained a known number of sachets in excess of the number required for 2 months supplementation at visits 0, 2, 5, 7 months. The children and their families were instructed to take Gln or placebo once per day in the morning mixed with water or yogurt. Compliance was assessed by the number of used and unused sachets returned at each visit following supplementation.

### Outcomes and follow-up

Based on the hypothesis that supplemental Gln would slow disease progression and therefore provide functional benefit, we compared change in walking speed after 4 months Gln versus placebo in ambulant DMD boys. The primary outcome measure was change in walking speed (m/s) measured in seconds taken to walk a specified distance (170 m) at a comfortable pace [27]. Secondary outcome measures included change in the following variables: 2-MWT (m) [27], [28], work (kcal), power (kcal/s), body composition, muscle mass (24-h urinary creatinine excretion), serum CPK (a non-specific indicator of sarcolemmal integrity), urinary excretion of 3-methyl-histidine/creatinine ratio (a marker of myofibrillar protein breakdown) [29] and oral nutrient intake. Incidence and severity of adverse events were assessed by reported signs and symptoms as well as physical examinations, vital signs and biochemical parameters. The children were evaluated every 2 months during period 1 (0, 2, 4 months) and period 2 (5, 7, 9 months) at one of the 3 clinical investigations centers (Paris, Poitiers or Lille). In total 6 visits were made by each child over the 9 month study.

Fasting blood samples were collected at 0, 2, 4, 5, 7, 9 months. Safety assessment including physical exam, vital signs measurements and biochemical parameters were performed at 0, 2, 4, 5, 7, 9 months and medical treatment was provided when required. Body composition was estimated by using monofrequency (50 kHz) bioelectrical impedance analysis (BIA; 101 Q; RJL systems, Clinton Township, MI or Quantum/S; Akern SRL, Pontassieve, Italy) at 0, 2, 4, 5, 7, 9 months and dual-energy X-ray absorptiometry (DXA; Lunar Corporation, Madison, WI or Hologic Discovery, Bedford, MA) at 4 and 9 months. Subjects were studied in the morning after an overnight fast and were instructed to void before being measured. Three consecutive 24 h urine collections were performed prior to each visit at 0, 2, 4, 5, 7, 9 months. Muscle mass was estimated from 24 h urinary creatinine excretion, assuming that 1 g per day of urinary creatinine excretion represents 20 kg of muscle mass [30]. Urinary 3-methyl-histidine concentration was measured using an automated unit and expressed relative to creatinine. Biochemistry was measured using an automated unit at 0, 2, 4, 5, 7, 9 months. Oral nutrient intake was assessed by a registered dietitian using a 2-d food record taken at visits 4 and 9 months. To ensure validity and quality of the data, monitoring was performed at each site during the trial and at study completion.

### Statistical analysis

Sample size estimates were based on the response to the primary outcome variable (walking speed in m/s). Previous data showed that walking speed for children with muscular dystrophy was 0.95±0.15 m/s [31]. For a crossover study, requiring a 10% increase in walking speed after 4 months Gln versus placebo, we calculated that 30 subjects were needed to have an 80% power to detect a difference at an α of 5%.

Data were double entered. Analysis was carried out by a blinded statistician using SAS version 9.1 (SAS Institute Inc., Cary, NC). Data are presented as medians (25% ; 75% quartiles (Q1 ; Q3)) or means (±standard deviation (±SD)) for continuous variables and as proportions (percentages) for categorical variables. Inter-group comparisons were performed using Student's *t* test for normally distributed data or Wilcoxon's rank test for non-parametric distributions and intra-group comparisons were performed using paired Student's *t* test or Wilcoxon's signed rank test as appropriate. The primary analysis of the data was on the primary outcome variable, change in walking speed. For a crossover design we evaluated treatment and order effects as well as the interaction. The effect of treatment with Gln was assessed using repeated-measures analysis of variance. The same approach was used to analyze the secondary outcome variables. Normality of residuals and absence of heteroscedasticity were verified, otherwise logarithmic or square root transformations were applied to the dependent variable. Significance was set at p<0.05.

## Results

### Participants

**Figure 1:**
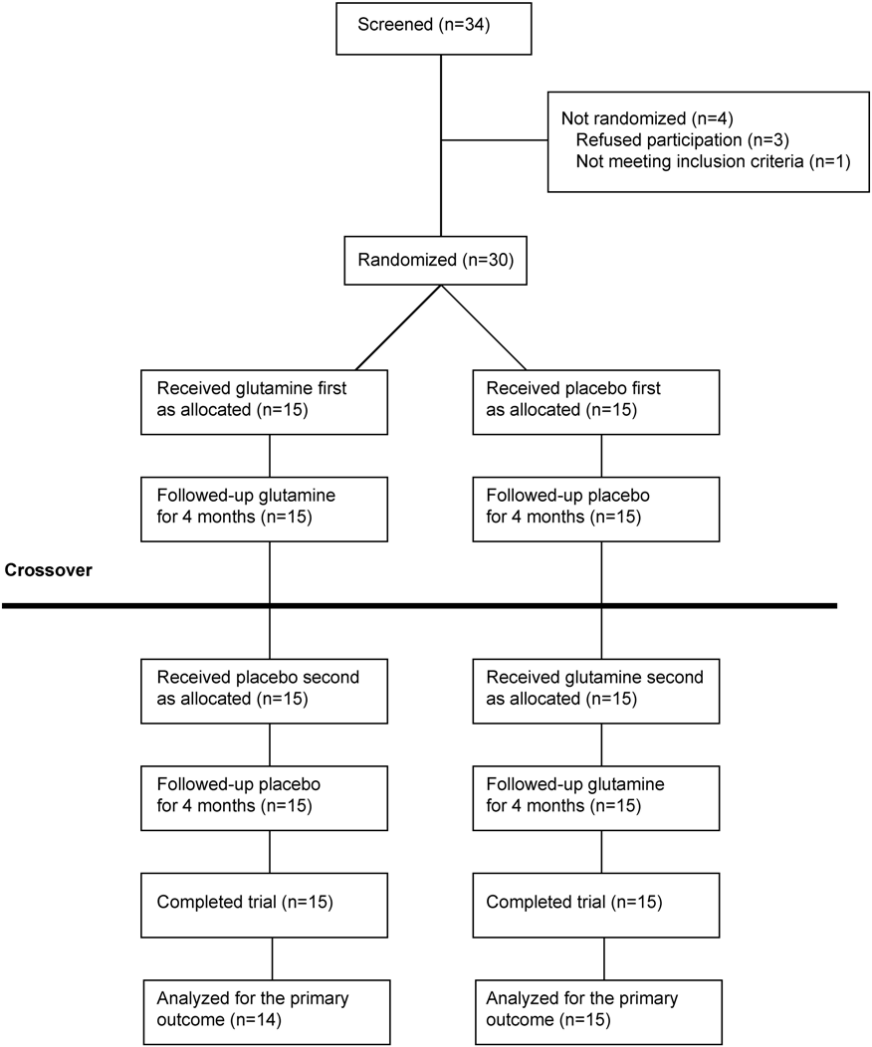
Flow of participants. Each 4 month intervention phase was separated by a washout period of at least 1 month.

Figure 1 shows the trial profile. The families of 34 boys were screened, of whom 30 boys aged 2 to 10 y were recruited. Agreement to participate was not received by 3 families and 1 boy did not meet one of the inclusion criteria because of body weight >60 kg. Of 30 boys randomized, all completed the study. One subject was not analyzed for the primary outcome, because he was unable to walk ≥170 m (secondary to gastroenteritis) at the 4 month visit during the placebo phase. Subjects were recruited between February 2006 and February 2007. The study was completed in November 2007. Baseline characteristics of the study population are presented in Table 1. Five boys were on corticosteroids during the trial and none were taking other potentially anabolic co-interventions or supplements (e.g. creatine).

**Table 1:** Baseline Characteristics.

	Subjects N = 30
Median age, y (Q1 ; Q3)	6.1 (4.6 ; 8.2)
Mean weight, kg (±SD)	19.7 (±6.0)
Mean weight, z score (±SD)	−0.49 (±1.1)
Mean height, cm (±SD)	112.7 (±14.5)
Mean height, z score (±SD)	−0.14 (±1.1)
Mean BMI, kg/m^2^ (±SD)	15.2 (±1.26)
Prepubescent, n (%)	30 (100%)
Corticosteroid treated, n (%)	5 (16.7%)
Median CPK, U/L (Q1 ; Q3)	13810 (9786 ; 17994)
Median 3MH/Creatinine ratio (Q1 ; Q3)	66 (54 ; 75)
Median muscle mass, kg (Q1 ; Q3)	4.1 (3.0 ; 5.1)
Median fat mass, % (Q1 ; Q3)	12.5 (4.7 ; 17.2)
Median fat free mass, kg (Q1 ; Q3)	16.2 (14.4 ; 19.2)
Median walking speed, m/s (Q1 ; Q3)	0.9 (0.7 ; 1.0)
Median 2-MWT, m (Q1 ; Q3)	103 (89 ; 120)

Q1 25% quartile; Q3 75% quartile; SD standard deviation; BMI body mass index; CPK creatine phosphokinase; 3-MH 3-methyl-histidine; 2-MWT 2-minute walk test.

### Outcomes and estimation

There was no functional improvement, based on the primary outcome measure (walking speed) during 4 months Gln treatment compared to placebo (Table 2). The same pattern was seen for 2-MWT, work and power, without any significant improvement after 4 months Gln treatment (Table 2). Notably the placebo group did not show deterioration of functional measures over a 4-month period (Table 2). Moreover, subjects overall did not show deterioration of functional measures over the course of the 9-month study. Mean walking speed (±SD) remained stable from 0.9 m/s (±0.2) at baseline to 0.8 m/s (±0.3) at 9 months (p = 0.78; paired samples *t* test). Similarly, the 2-MWT did not differ from baseline (103.9 m (±20.4)) to the end of study, at 9 months (98.9 m (±28.1)) (p = 0.39; paired samples *t* test).

**Table 2:** Primary and Secondary Outcome Measures.

	Placebo	Glutamine
Median walking speed, m/s (Q1 ; Q3) †	n = 29	n = 30
Pre	0.8 (0.7 ; 1.0)	0.9 (0.7 ; 1.0)
Endpoint (4 mo)	0.8 (0.6 ; 1.0)	0.9 (0.7 ; 1.0)
Change at 4 mo	0.0 (−0.1 ; 0.1)	0.0 (−0.1 ; 0.1)
Median 2-MWT, m (Q1 ; Q3) †	n = 29	n = 30
Pre	100.0 (86.0 ; 120.9)	105.5 (89.9 ; 115.2)
Endpoint (4 mo)	101.0 (81.6 ; 113.4)	101.5 (87.0 ; 109.0)
Change at 4 mo	2.0 (−16.2 ; 13.3)	−2.8 (−10.0 ; 2.3)
Median work, kcal (Q1 ; Q3) †	n = 29	n = 30
Pre	16.0 (10.9 ; 25.5)	17.0 (11.2 ; 27.1)
Endpoint (4 mo)	16.3 (12.9 ; 24.4)	18.2 (9.3 ; 24.9)
Change at 4 mo	−1.1 (−7.1 ; 6.3)	0.7 (−5.1 ; 2.0)
Median power, watt (Q1 ; Q3) ‡	n = 29	n = 30
Pre	157 (117 ; 193)	170 (108 ; 195)
Endpoint (4 mo)	152 (113 ; 195)	159 (118 ; 201)
Change at 4 mo	11 (−13 ; 23)	10 (−17 ; 20)
Median fat mass, % (Q1 ; Q3) †	n = 27	n = 29
Pre	14.3 (6.2 ; 20.4)	11.3 (5.0 ; 18.8)
Endpoint (4 mo)	14.2 (8.2 ; 22.2)	13.8 (9.9 ; 21.8)
Change at 4 mo *	1.2 (−1.6 ; 2.3)	1.9 (−0.8 ; 4.9)
Median fat free mass, kg (Q1 ; Q3) †	n = 27	n = 29
Pre	15.9 (14.0 ; 19.3)	16.2 (14.6 ; 20.1)
Endpoint (4 mo)	17.4 (14.9 ; 19.0)	16.6 (14.7 ; 19.6)
Change at 4 mo **	0.7 (0.2 ; 1.2)	0.4 (−0.2 ; 0.7)
Median muscle mass, kg (Q1 ; Q3) †	n = 27	n = 26
Pre	3.9 (3.1 ; 5.2)	3.8 (2.4 ; 4.8)
Endpoint (4 mo)	3.6 (2.3 ; 4.2)	3.2 (2.7 ; 4.1)
Change at 4 mo	−0.3 (−1.5 ; 0.3)	−0.7 (−1.1 ; −0.1)
Median CPK, U/L (Q1 ; Q3) †	n = 30	n = 30
Pre	13530 (9140 ; 18186)	12010 (10160 ; 17289)
Endpoint (4 mo)	12740 (8380 ; 18350)	10430 (9160 ; 15929)
Change at 4 mo	−795 (−3402 ; 3332)	−2010 (−4984 ; 1340)
Median 3-MH/creatinine (Q1 ; Q3) †	n = 25	n = 24
Pre	72.0 (61.5 ; 78.0)	68.3 (54.0 ; 79.0)
Endpoint (4 mo)	71.9 (61.6 ; 81.0)	67.0 (57.0 ; 76.0)
Change at 4 mo	−0.7 (−7.2 ; 8.0)	−4.8 (−16.7 ; 8.8)

* *p*<0.05 (glutamine vs placebo, repeated-measures analysis of variance without order effect or interaction).

** *p*<0.05 (glutamine vs placebo, repeated-measures analysis of variance with order effect).

† Logarithmic transformation.

‡ Square root transformation.

Q1 25% quartile; Q3 75% quartile; CPK creatine phosphokinase; 3-MH 3-methyl-histidine; 2-MWT 2-minute walk test.

Over the 9-month trial, there was a significant increase in fat free mass as measured by BIA and fat mass percentage as measured by BIA and DXA. Median (Q1 ; Q3) fat free mass increased from 16.2 kg (14.4 ; 19.2) at baseline to 17.4 kg (15.0 ; 19.5) at 9 months (p<0.0001; Wilcoxon's signed rank test) and percentage fat mass increased from 11.3% (4.7 ; 17.2) at baseline to 16.1% (9.9 ; 21.8) at 9 months (p<0.0001; Wilcoxon's signed rank test) in subjects overall. The increase in fat free mass was significantly less with 4 months Gln treatment versus placebo (Table 2) and an order effect was observed (p = 0.001). In turn, the increase in percentage fat mass as measured by BIA was significantly greater with Gln treatment (Table 2). Similarly, median percentage fat mass (Q1 ; Q3) measured by DXA was 20.2% (14.1 ; 27.4) after 4 months Gln treatment and differed from placebo (18.5% (14.1 ; 28.8)) (p = 0.03; variance analysis, square root transformation). Median muscle mass (Q1 ; Q3) estimated by 24 h urinary creatinine excretion decreased from 4.1 kg (3.0 ; 5.1) at baseline to 3.5 kg (2.6 ; 4.1) at 9 months (p<0.0001; Wilcoxon's signed rank test). However, the change in muscle mass with 4 months Gln treatment was not different from placebo (Table 2). Consistent with muscle mass, markers of myofibrillar protein breakdown (3 methyl-histidine/creatinine ratio in urine) and serum CPK were not affected by Gln treatment compared with placebo (Table 2). Furthermore, the 24 h excretion of 3 methyl-histidine in urine was not affected by 4 months Gln treatment (p = 0.25).

The families of 15 subjects (50%) considered the child's condition improved during the 4 months Gln treatment, whereas 12 families (40%) found that their child's condition improved during the placebo phase. The families of 3 subjects (10%) found the child's condition equivalent in both phases.

There was no significant difference in oral nutrient intake during 4 months Gln treatment compared with 4 months placebo, as assessed by median (Q1 ; Q3) total daily energy intake (1526 kcal (1399 ; 1758) for the placebo group compared with 1529 kcal (1331 ; 1766) for the Gln group) or percentage daily kcal from protein (16% (15 ; 19) for the placebo group compared with 17% (14 ; 19) for the Gln group). Compliance as assessed by the number of used and unused sachets returned was also similar between Gln treatment and placebo.

### Safety and Tolerability

Gln and placebo were safe and well-tolerated by most of the children with no significant difference in side-effect profiles or biochemical parameters between groups. Adverse events reported during the 4-months Gln and placebo treatment periods included: gastroenteritis (Gln, n = 1; placebo, n = 2), urticaria (Gln, n = 1; placebo, n = 0), allergic reaction (Gln, n = 0; placebo, n = 1), nervousness (Gln, n = 1; placebo, n = 0), rhinitis (Gln, n = 0; placebo, n = 1) and pharyngitis (Gln, n = 0; placebo, n = 1).

## Discussion

### Interpretation

Although experimental data in *mdx* mice [9], [11] and short term clinical studies on protein metabolism [19], [24], suggest a role for Gln in the treatment of DMD, the present randomized crossover trial did not show improved muscle function when oral Gln administered for 4 months was compared to placebo in ambulatory DMD boys. Only 1 previous RCT tested the efficacy of Gln in DMD boys [17], using a parallel trial to compare 6-months oral Gln (0.6 g/kg/d) (n = 19) with placebo (n = 16) and showed no significant effects on manual or quantitative measures of muscle strength. This previous trial showed significantly less deterioration in timed functional tests in the younger subgroup (<7 y), however the significant age-related results were based on an unplanned subgroup analysis in a small group of DMD boys [17].

In the present trial, we assessed muscle function by walking speed. We employed a functional measure as the primary outcome, because timed function testing is a clinically relevant trial endpoint in DMD [32]. Previous data suggest that timed functional tests are reliable measures that should be used in multicenter trials in ambulant DMD children to obtain maximum power and sensitivity [32], [33]. Few trials have demonstrated a functional benefit in DMD. A previous RCT of prednisone in DMD showed improvement in function to peak at 3 months [2]. The present trial however did not detect arrest of disease progression or minimal improvement in function with 4 months Gln over placebo. We used timed functional testing which is more reliable [32] and sensitive to determine the extent of disease progression in DMD [33] than muscle force measurement. Furthermore, timed walk tests have been shown to have high test-retest reliability in a range of patients with neurological disorders (intraclass correlation coefficient of 0.97) [27] and in children with cystic fibrosis (coefficient of variation of 2.6%) [34]. Our findings that functional measures did not deteriorate during the 4-month placebo phase, or over the course of 9 months, were not as expected [35]. Based on natural history data [35], the present crossover trial was sufficiently powered to detect a 10% increase in walking speed after 4 months Gln compared to placebo. We however did not consider that children respond more strongly to placebo in RCTs than adults do, according to a recent meta-analysis in drug resistant partial epilepsy [36]. Thus the greater placebo effect in children could have narrowed the expected effect size of Gln treatment. Furthermore, the finding that the families of 40% of subjects reported that their child's condition improved during the placebo phase (versus 50% during Gln treatment) also supports a placebo effect by proxy, which has been suggested in younger populations, since parents play an important role in reporting the outcome of their affected child [36]. Future trials might consider including a subject group receiving no intervention or a longer baseline period, to measure the placebo effect in DMD children.

In the present trial, urinary excretion of 3-methyl-histidine/creatinine ratio was not affected by 4 months Gln, thus the acute anti-proteolytic effect of Gln may be transient [19], [24]. Because subjects were not on a meat-free diet prior to urine collection, this analysis is limited. However, there were no differences in oral protein intake during Gln treatment compared with placebo. Gln treatment also did not affect serum CPK or muscle mass (estimated by urinary creatinine excretion), which showed a similar decrease in the placebo group. Whereas the present study and previous reports [37] showed that urinary creatinine excretion progressively decreases with muscle wasting in DMD, we observed an increase in fat free mass and percentage fat mass (measured by BIA). This is not surprising, since BIA estimates total body water, composed of intracellular and extracellular water. Although reduced muscle mass may contribute to decreased total body water, the correlation between the 2 variables was reported to be weak [38], [39]. In DMD, muscle mass correlates with intracellular water and total body potassium, which decline with age and reflect progression of disease [38]. At variance, exchangeable sodium (or extracellular water) as well as total body water increase with growth in DMD [39]. Furthermore, the relative contribution of fat free mass to body weight in DMD boys is 73% (versus 84% in control children), which is greater than expected for a group of patients with an estimated muscle mass relative to body weight of only 12% (versus 39% in control children) [40]. This discrepancy might reflect the replacement of potassium-rich muscle by potassium-poor hydrated connective tissue or an increase in extracellular water in addition to fat [38], [39], producing the pseudo-hypertrophy well-recognized clinically [39]. Thus, the BIA method can provide estimates of fat free mass (or total body water) in DMD [41], but it cannot provide estimates of muscle mass. The 9% increase in fat free mass during the placebo phase was significantly higher than that observed after Gln treatment (+2%) which led to a greater increase in percentage fat mass with Gln treatment. Because the treatment effect was on the borderline of significance, it is possible that this may have been a chance finding. Alternatively, it is possible that the increase in fat free mass during the placebo phase reflects the natural progression of disease. We also observed an order effect on fat free mass, which could result from a difference in disease progression between the first and second phase of the study or a response to treatment that varies with time.

### Generalizability

Although the criteria that we used to select participants were simple and as broad as possible to be generalized to DMD patients, Gln may also have different effects depending on the stage of the disease process [17]. Inclusion of younger children in which measures of function are difficult to assess objectively [32], could explain some of the variability in functional measures. Alternatively, early treatment, before the pathology manifests are expected to be the most beneficial [42]. Thus younger children may be more likely to respond to treatment and may represent a subgroup for which Gln may prove beneficial. Of interest, subgroup analysis revealed a differential decline in functional measures in those taking corticosteroids (versus those on no corticosteroids). For example in boys taking corticosteroids (n = 5), mean walking speed (±SD) decreased from 1.0 m/s (±0.1) at baseline to 0.7 m/s (±0.2) at 9 months (p<0.05; paired samples *t* test), whereas those not on corticosteroids (n = 25) showed no deterioration in function over time (baseline: 0.8 m/s (±0.2) versus 9 months: 0.8 m/s (±0.3)), similar to the cohort as a whole. There was also a significant effect of Gln treatment on functional measures in boys taking corticosteroids (p<0.05; variance analysis, logarithmic transformation). Specifically, boys taking corticosteroids showed a significant decline in median walking speed (Q1 ; Q3) during the placebo phase (pre: 1.0 m/s (0.7 ; 1.0) versus 4 months: 0.6 m/s (0.6 ; 0.7)), whereas walking speed remained stable when corticosteroid-treated boys received Gln treatment for 4 months (pre: 0.9 m/s (0.9 ; 1.0) versus 4 months: 1.0 m/s (0.8 ; 1.0)). Although the findings must be interpreted with caution, because they derive from an unplanned analysis in a small subgroup of boys, they might suggest a rationale for Gln supplementation in conjunction with corticosteroid therapy [43], [44], which needs to be investigated.

Because of the heterogeneity in patients who have this disease, we used a crossover design where all subjects served as their own controls. While sharing the strengths of a RCT, the efficient design also permitted us to use a smaller sample size versus a parallel trial, which is particularly suitable for rare diseases. Although a problem with the crossover trial is carry-over, we used a washout period. And given that whole body Gln turnover is high in adults [26] and even higher in children [45], with complete renewal in <24 h [20], we could assume minimal or no carry-over after 1 month. Furthermore, we found no significant effects of intervention sequence on the relative treatment responses. Among constraints, our study required longer intervention and follow-up, since all subjects received placebo. On the other hand, all children received a potentially beneficial treatment, which is particularly interesting for chronic non-curable diseases where patients have few medical options. The study demonstrates the feasibility of a 9-month crossover trial, since all subjects completed the trial with good compliance. Although this was not a safety study and was not powered to detect significant side effects, the data imply that side effects were minimal when Gln was administered over 4 months and no subjects withdrew. Safety of supplementation for longer durations is beyond the scope of this study.

### Overall evidence

The present study provides important information to guide the practice of medicine and is of broad interest to several disciplines, including child neurologists, movement disorder specialists and nutritionists. Apart from corticosteroids [2], treatment options are limited. DMD patients and their families often resort to alternative therapies (e.g. nutrition supplements) that may be potentially beneficial. However, the physician/neurologist has little scientific evidence to base their judgement, given the few methodologically sound RCTs on nutritional therapies in DMD [16]–[18]. Although apparently safe, 4 months Gln supplementation did not prove to be beneficial compared to placebo, based on muscle mass or function. Thus, the present RCT cannot support its routine use in this population as a whole. Better targeting of specific subgroups is necessary to fully evaluate the presence or absence of benefits. More precise knowledge is also needed regarding the response to placebo in DMD children. The present trial has implications for the design of future clinical trials in DMD children or other pediatric conditions.

## Supporting Information

Appendix S1 List of the people who participated in this trial, by site.
(0.02 MB DOC)

Checklist S1 CONSORT checklist
(0.06 MB DOC)

Protocol S1 Trial Protocol (in French)
(0.35 MB DOC)

Statement S1 Statement from the ethics committee indicating their approval of the research (in French)
(1.19 MB PDF)

Form S1 Sample of a patient (parent) consent form (in French)
(0.11 MB PDF)

Form S2 Sample of a patient (children) assent form (in French)
(0.06 MB PDF)
