# Supplementary material for: Lack of Functional Benefit with Glutamine versus Placebo in Duchenne Muscular Dystrophy: A Randomized Crossover Trial
Source: PLoS One. 2009 May 6;4(5):e5448. doi: 10.1371/journal.pone.0005448 (PMC2673684; doi:10.1371/journal.pone.0005448)
Supplement: Appendix S1 — List of the people who participated in this trial, by site. (0.02 MB DOC) [file pone.0005448.s001.doc]

**APPENDIX**

The following people participated in this study (by site): INSERM CIC 9202, Hôpital Robert Debré: André Denjean, MD, Evelyne Jacqz-Aigrin, MD, Fanny Thourer, Ying Wang, MD, Christelle Guimber, Maria Ferraz, Véronique Berruer, Christine Samy, Alicia Lefevre, Stéphanie Meslin, Christel Daubrosse ; Unité d’Epidémiologie Clinique, Hôpital Robert Debré: Corinne Alberti, MD, Etienne Lucas, Anne Gaudelet; Biochimie, Hôpital Robert Debré: Odile Rigal, Didier Chevenne; INSERM CIC 9301 CHR&U de Lille: Jean-Marie Cuisset, MD, Frédéric Gottrand, MD, Christian Libersa, MD, Laurent Béghin, Charlotte Delcroix, Fernande Noté, Catalina Iliescu, MD, Patrick Rasoamanana, MD, Gwenaelle Maton, Marie Castéra; INSERM CIC 802 CHU de Poitiers: Régis Hankard, MD, Elise Mok, Guy Letellier, MD, Anne-Sophie Gourgues, Elodie Rogeon, Brigitte Lafaille, Marie-France Bourin, Géraldine Martineau, MD, Patricia Christin, MD; Hôpital Necker Enfants Malades: Louis Viollet, MD; CH de Rochefort: Denis Graber, MD; CH de Saintes: Youssef Wardi, MD; CHU de Grenoble: Marie-Christine Commare, MD; AGEPS: Florence Barat, Annick Tibi; Groupe Hospitalier Bichat-Assistance Publique-Hôpitaux de Paris: Caroline Quintin, Séverine Lopez, Hélène Picard; Département de la Recherche Clinique et du Développement-Assistance Publique-Hôpitaux de Paris : Christophe Aucan
