# Supplementary material for: Lack of Functional Benefit with Glutamine versus Placebo in Duchenne Muscular Dystrophy: A Randomized Crossover Trial
Source: PLoS One. 2009 May 6;4(5):e5448. doi: 10.1371/journal.pone.0005448 (PMC2673684; doi:10.1371/journal.pone.0005448)
Supplement: Protocol S1 — Trial Protocol (in French) (0.35 MB DOC) [file pone.0005448.s003.doc]

**Titre :** Effet de la prise orale de glutamine sur la fonction et la masse musculaire dans la myopathie de Duchenne de Boulogne

**Mots-clefs :** Pédiatrie – Maladie Rare – Glutamine – Essai Thérapeutique – Nutrition

**Etude avec bénéfice individuel**

**Etude randomisée, en « cross over » contre placebo, en double aveugle**

**Nombre de patients :** 30

**Durée de l’étude :** 9 mois

**Période d’inclusion :** 2 ans

**Durée totale d’étude :** 3 ans

**Projet Inter-CIC tricentrique**

**Investigateur Principal :**

- Régis Hankard Département Médico Chirurgical de Pédiatrie et Centre de Recherche Clinique, CHU de Poitiers, 2 rue de la Milétrie, 86021 Poitiers Cedex
  - Tel : 05 49 44 49 18 Fax : 05 49 44 40 16
  - Mail : [r.hankard@chu-poitiers.fr](mailto:r.hankard@chu-poitiers.fr)

**Collaborations :**

**Paris**

Hôpital Robert Debré

- Corinne ALBERTI (Unité d’Epidémiologie Clinique)
- Olivier BOURDON (Pharmacie Hospitalière)
- André DENJEAN (Physiologie–Explorations Fonctionnelles

Hôpital Necker Enfants Malades

- Isabelle DESGUERRE (Service du Métabolisme, Nutrition)

Hôpital St-Vincent de Paul

- Michèle MAYER (Service de Neuropédiatrie)

## Poitiers

CHU de Poitiers

- Gilles KEMOUN (Service de Médecine Physique et Réadaptation)

# Lille

Hôpital Roger Salengro

- Jean Marie CUISSET (Service de Neurologie Infantile-Maladies Inféctieuses)

Hôpital Jeanne de Flandre

- Frédéric GOTTRAND (Service d’Hépato Gastro Entérologie)

Hôpital Cardiologique

- Christian LIBERSA (Centre d’Investigation Clinique)

**Résumé**

La glutamine inhibe la dégradation protéique corporelle totale chez l’enfant atteint de myopathie de Duchenne de Boulogne (MDB)**.** Cet effet observé après 5h de prise orale de glutamine est retrouvé lorsque la glutamine est donnée pendant 10 jours. Il est spécifique car il n’est pas reproduit par un mélange iso-azoté d’acides aminés (effet spécifique). Cette étude **tricentrique nationale** (Paris, Poitiers et Lille) **randomisée**, en **double aveugle** et en **cross-over** glutamine **contre placebo** évalue le **bénéfice fonctionnel** de la prise de glutamine orale sur deux périodes de 4 mois séparées d’une **fenêtre thérapeutique** d’1 mois. L’objectif d’améliorer la vitesse de marche de 10% (glutamine vs placebo) porte le nombre de patients à inclure à **30 enfants**. Les enfants seront vus tous les 2 mois (M0,M2,M4,M5,M7,M9) dans les **Centres d’Investigation Clinique** de Paris-Robert Debré et du CHRU de Lille et dans le service de Pédiatrie du CHU de Poitiers. Le critère principal de jugement sera la vitesse de marche sur un parcours standardisé. Les critères secondaires incluent le travail (Kcal) et la puissance (Kcal/s) liées à l’effort, la composition corporelle (impédancemétrie et absorptiométrie biphotonique), la masse musculaire estimée par la créatininurie des 24h, l’évolution des CPK et l’excrétion de 3-MH (index de dégradation protéique) ainsi que le suivi de paramètres biochimiques (ionogramme, glycémie à jeun, transaminases, insulinémie, IgfI, Igf-BPI). La mise en évidence d’un bénéfice fonctionnel imposera d’évaluer l’administration de glutamine sur de plus longues durées (le plus tôt possible après le diagnostic), chez des enfants déjà lourdement handicapés et dans d’autres pathologies chroniques associées à un catabolisme protéique musculaire accru (situations de stress, corticothérapie prolongée, …). Dans la MDB elle contribuera à mener les enfants à la thérapie génique dans les meilleures conditions.

**Justification de l’étude**

La glutamine stimule l’anabolisme protéique. Chez le sujet sain, elle stimule la synthèse protéique (1). En pathologie humaine, elle inhibe la dégradation protéique. Chez l’**enfant** atteint de myopathie de Duchenne de Boulogne (MDB), **maladie rare** qui se caractérise par une perte progressive et massive de la masse musculaire, nous avons montré que :

1. la prise orale de glutamine **inhibait la dégradation protéique** et la synthèse endogène de glutamine (2). Mais cet effet était observé sur une courte période (5h) et rien n’indiquait qu’il persistait avec le temps
2. l’inhibition de la dégradation protéique corporelle totale **persiste** lorsque la glutamine est prise par **voie orale** pendant 10 jours consécutifs (3) (Etude de promotion AP-HP, terminée en 2002).
3. L’effet est **spécifique** car non reproduit chez le sujet sain par un apport iso-azoté d’un autre acide aminé, la glycine (1) et dans la myopathie par un apport iso-azoté d’un mélange d’acides aminés reproduisant la composition des protéines de l’œuf (3) (Etude de promotion AP-HP, terminée en 2002).

Dans un travail récent réalisé chez la souris mdx, un modèle animal de myopathie de Duchenne de Boulogne, la glutamine a été identifiée comme présentant un intérêt thérapeutique potentiel car elle améliore les performances des animaux (4).

Les mécanismes d’action sont encore mal connus :

1. Dans un modèle de foie isolé perfusé, l’anabolisme protéique observé avec la glutamine est associé à un **gonflement cellulaire** (5). Une diminution du volume intracellulaire induit des effets inverses. Le volume cellulaire apparaît ainsi comme un signal métabolique capable de modulation transcriptionnelle pour des facteurs de transcription c-jun, des enzymes du cycle de l’urée (ornithine décarboxylase, arginino-succinate synthétase, des enzymes de la néoglucogénèse (PEPCK), des protéines (β-actine, tubuline)(6).
2. La synthèse endogène de glutamine (principalement musculaire) est modulée par l’apport de glutamine exogène. Nous avons en effet observé in vivo chez l’adulte sain et l’enfant myopathe que la prise de glutamine inhibait la production endogène corporelle totale de glutamine et permettait l’économie d’acides aminés engagés dans cette voie de synthèse (2, 7). L’absence de glutamine dans le milieu de culture stimule la glutamine synthétase sans effet sur la glutaminase dans un modèle de myoblastes L6 de rat (8). La **glutamine synthétase** (GS) serait donc une étape clef de la régulation du métabolisme de cet acide aminé. Récemment les travaux de Souba et collaborateurs ont montré que la glutamine était responsable d’une modulation transcriptionnelle mais aussi post transcriptionnelle de la GS. En effet l’augmentation des mARN de GS ne s’accompagne d’une augmentation de la GS que lorsque la concentration intracellulaire de GLN est basse (9).
3. La glutamine module la concentration de protéines HSP (HSP70, Hème oxygénase 1 =HSP32)(10) . L’augmentation de HSP 70 dans un modèle de myotubes prolonge la demi-vie de protéines myofibrillaires (11) .
4. La carence en certains acides aminés dont la glutamine stimule l’expression de CHOP (GADD153) dont l’expression module certains facteurs de transcription nucléaires (C/EBP,…). Cette régulation a lieu à la fois au niveau transcriptionnel et post-transcriptionnel (12, 13). L’expression de l’asparagine synthétase (AS) est elle aussi stimulée par la carence en glutamine. Les gènes de CHOP et AS partagent une même région promotrice responsable de la modulation de l’expression par les acides aminés (AARE : Amino acid response element). D’autres gènes sont aussi modulés par les acides aminés (IGFBP1, arginosuccinate synthétase, C/EBPα, C/EBPβ, β-actine, ubiquitine c, apoB100, calreticuline, Jun, Myc, …)(14).
5. La glutamine active certaines Mitogen Activated Protein Kinases (MAPK) notamment ERK et JNK qui activent les facteurs de transcription AP-1. Ce mécanisme pourrait rendre compte de la stimulation de la prolifération cellulaire observée avec la glutamine notamment au niveau intestinal (15). Dans le foie, le gonflement cellulaire active cette voie (16)
6. Enfin la biodisponibilité des acides aminés intervient dans les mécanismes translationnels directement responsables de la synthèse protéique (synthèse du ribosome 43S et sa régulation). Ces étapes seraient contrôlées par le système mTOR. Des travaux récents suggèrent que ce même système mTOR contrôlerait aussi les systèmes de dégradation protéique (17).

En clinique, la glutamine a été étudiée dans le **stress en milieu chirurgical, de réanimation ou lors d’une greffe de moelle** (18-31), chez le **prématuré** (32-36), dans **l’insuffisance intestinale** (37-39). Par voie intraveineuse elle est souvent administrée sous forme de **di-peptides** car elle est instable en solution (18-20, 24, 27, 30). Certaines études concernent la **voie entérale** (23, 26, 31, 32, 35, 36, 40, 41). La glutamine **améliore la balance azotée** (18-20, 24, 25, 27-29, 32, 34, 40, 41), **diminue le** **taux de complications notamment infectieuses** (21, 22, 28-31, 33, 35), la **durée d’hospitalisation** (18, 20, 21, 23, 28, 29, 33, 35) et donc le **coût de prise en charge** (21, 23, 29, 33, 36).

En physiologie humaine la glutamine : est l’acide aminé libre le plus abondant dans l’organisme (42), est à l’interface du métabolisme du glucose et des lipides et constitue un substrat majeur de la néoglucogenèse (43, 44), bloque la lipolyse et la cétogenèse du jeûne (45), est un substrat majeur pour les cellules intestinales chez l’animal (46),chez l’adulte et l’enfant (47, 48) et les tissus à renouvellement rapide en général.

**But de l’étude**

Le but de cette étude est de mettre en évidence, pour la première fois dans cette indication, un **bénéfice clinique** associé à la prise de glutamine. Elle fait suite à une série d’études qui ont exploré l’effet de la glutamine sur le métabolisme protéique dans la myopathie de Duchenne de Boulogne.

**Objectif principal**

1. Comparer la vitesse de marche d’enfants myopathes recevant dans un ordre tiré au sort de la glutamine ou un placebo

**Objectif secondaire**

1. Evaluer l’effet de la glutamine sur la masse musculaire, des indices du métabolisme protéique et la compostion corporelle.

**Hypothèse**

La glutamine freine la progression de la maladie en inhibant la dégradation protéique. L’enfant préserve ainsi son capital musculaire et la progression du handicap est ralentie. En pratique, sa vitesse de marche doit rester stable voire s’améliorer pendant la période où il reçoit de la glutamine et diminuer en période contrôle.

**Critère principal de jugement**

1. Vitesse de marche sur un parcours standardisé.

**Critères secondaires**

1. Travail et puissance associées à l’effort de marche
2. Composition corporelle déterminée par absorptiométrie biphotonique et par impédancemétrie monofréquence
3. Catabolisme musculaire évalué par la concentration plasmatique de Créatine Phospho Kinase (CPK)
4. Masse musculaire estimée par créatininurie des 24h
5. Dégradation protéique estimée par l’excrétion de 3-Méthyl-Histidine urinaire (3-MHu) et du rapport 3-MHu/créatininurie (3-MH/Créa.u)

###### Population et méthodes

**Schéma expérimental**

Cette étude est **prospective**, **randomisée contre placebo** en « **cross-over** », en **double aveugle**. Elle inclut deux périodes de 4 mois : 1) une période « glutamine »  où l’enfant recevra 0,5g/kg/j de glutamine en une prise le matin et 2) une période « contrôle » où il recevra un placebo de même poids et aspect. Les deux périodes seront distantes d’un mois, période de **fenêtre thérapeutique ;** l’ordre sera tiré au sort. L’enfant sera vu 7 fois (Visite d’inclusion, M0,M2,M4,M5,M7,M9) dans un **Centre d’Investigation Clinique (CIC)**. La visite d’inclusion aura lieu au minimum 1 semaine et au maximum 2 mois avant M0. Les examens réalisés figurent dans le tableau I.

**Critères d’inclusion**

- Myopathie de Duchenne de Boulogne (diagnostic porté par le neurologue de la consultation multidisciplinaire)
- Périmètre de marche ≥ 170 m
- Absence d’insuffisance rénale ou hépatique
- Consentement de participation signé

**Critères de non inclusion**

- Dépendance d’un fauteuil roulant
- Poids supérieur à 60 kg
- Décision de l’enfant ou des parents
- Toute Chirurgie (membres inférieurs, rachis, …) programmée dans l’année qui suit la visite d’inclusion

**Critères de sortie d’étude**

- Décision de l’enfant ou des parents

**Méthodes**

**Vitesse de marche (M0,M2,M4,M5,M7,M9)**

Elle est mesurée sur une piste plane de 85 m de long en intérieur. L’enfant marche à sa vitesse de confort  sur une distance de 170 m. Si l’enfant ne peut marcher 170 m, la distance parcourue et le temps sont notés (marquage au sol et chronométrage). L’analyse portera sur la différence observée entre le début et la fin de la période considérée.

Les résultats seront traduits en énergie (E) ou travail:

**E (Kcal) = m.d.g / 4,18**

avec m = masse en kg, d = déplacement en m, g = accélération de la pesanteur (9.81 m/s^2^) et 1 kcal = 4,18 kJ.

Exprimés par unité de temps, le travail a la dimension d’une puissance (P en Watt ou J/s) :

**P = E . 4,18 / t**

avec t= temps en sec.

**Mesure de la composition corporelle par impédancemétrie (M0,M2,M4,M5,M7,M9)**

L’impédancemétrie monofréquence (RJL BIA 101-Q) est une méthode simple, rapide (<5’), non invasive qui permet d’estimer la composition corporelle. Les mesures sont réalisées dans des conditions standardisées (le matin entre 7 et 10h, à jeun, vessie vidée, en position allongée depuis 10’, sans contact avec une surface métallique). L’appareil mesure la résistance corporelle opposée à un courant de très faible intensité (800µA) et de 50kHz. Les équations utilisées sont celles de Houtkooper et coll (49) que nous avons comparées avec une technique de référence dans la MDB (50).

**Mesure de la composition corporelle par DXA (M4,M9)**

Ces mesures seront réalisées à chaque fin de période d’étude avec un appareil Lunar®. Le principe de la mesure est d’utiliser la différence d’atténuation de deux rayonnements d’énergie différente pour évaluer la masse maigre et grasse du corps entier. Une mesure dure environ 10’ et l’exposition est de l’ordre du 1/10 d’une radiographie pulmonaire standard.

**Dosages biologiques**

***Dosages sanguins (M0,M2,M4,M5,M7,M9)***

Ils incluent le dosage de CPK, un ionogramme sanguin avec urée et créatinine, un bilan hépatique (TGO, TGP, PA), des dosages hormonaux (glycémie à jeun et insuline à jeun, IgF-I, IgFBP1).

La concentration plasmatique de CPK est un paramètre de jugement de l’effet sur l’évolution de la maladie. Elle est initialement très élevée en raison de la myolyse. Elle diminue ensuite de 15-20% par an (51).

***Dosages urinaires (M0,M2,M4,M5,M7,M9)***

Le dosage de la créatininurie des 24h permet une estimation de la masse musculaire (1g/j pour 20kg de muscle). Elle est moyennée sur une durée de recueil d’urine de 3 j. Dans les études précédentes que nous avons réalisées, la durée d’administration de la glutamine était trop courte pour mettre en évidence un effet sur la masse musculaire. Dans une autre étude de 6 mois, le bénéfice fonctionnel associé à la prise de corticoïdes s’accompagnait d’une augmentation de 35% de la créatininurie contre 6% de diminution dans le groupe contrôle (52).

Le dosage du rapport 3-MHu/Créa.u est un reflet de la dégradation des protéines myofibrillaires. Il contribue à documenter une inhibition de la dégradation protéique induite par la glutamine

**Enquête diététique (M4, M9)**

Elle est réalisée par une diététicienne DE à partir d’un carnet de recueil sur 2 jours (1 jour de WE , un jour de semaine). Les données seront exploitées sur le logiciel Régal-Micro(M Feinberg, Ed. Tec&Doc et Inra).

**Analyse statistique**

**Gestion des données et statistique :**

- **Justification du nombre de patients**

L’hypothèse nulle H_0_ repose sur l’absence d’autre effet sur la vitesse de déplacement en centimètre par seconde mesurée à 4 mois qu’un effet de la glutamine ingérée durant cette même période.

On pose

- *N’*  représente le nombre de sujets dans un « cross-over » au sein duquel on effectuera 2*N’* mesures de la vitesse de déplacement.
- *N*  représente le nombre de sujets théoriques qu’on aurait calculé dans un essai en deux groupes parallèles
- $\rho'$est le coefficient de corrélation entre la 1^ère^ et la 2^ème^ mesure chez un même sujet

Le nombre de sujets à inclure est de $N'=\frac{N}{2}x(1-\rho')$

On peut estimer que la vitesse de déplacement est de 95 cm/s avec une déviation standard de 15 cm/s (53). On considère que l’ingestion de glutamine aura pour effet d’augmenter la vitesse de déplacement de 10%. En situation de 2 groupes parallèles avec un risque alpha de 5% et une puissance de 80%, il faudra inclure 74 patients. Nous n’avons pas d’idée a priori sur l’estimation de $\rho'$ mais plusieurs calculs d’effectifs sont effectués :

| $\rho'$ | Nombre de sujets nécessaire |
| --- | --- |
| 0  0.1  0.2  0.3  0.4  0.5 | 37  33  30  26  22  19 |

Compte-tenu des possibilités de recrutement on prévoit d’inclure 30 patients.

- **Stratégie d’analyse des données collectées**
- **Analyse descriptive**

Les données qualitatives seront décrites sous forme de fréquence et pourcentages, les quantitatives sous forme de moyenne (déviation standard) si la distribution suit une loi normale, sous forme de médiane (quartiles) sinon.

- **Analyse explicative**

L’analyse principale sera effectuée sur la vitesse de déplacement (mesurées en cm/s) sur les patients qui auront complété les deux périodes.

Soit le tableau suivant :

|  | Période | |
| --- | --- | --- |
|  | Temps 1 | Temps 2 |
| S1 = Placebo puis glutamine | X_i1_ | X_i2_ |
| S2 = glutamine puis placebo | X_j1_ | X_j2_ |

S1 : séquence 1 S2 : séquence 2

X_i1_ est la mesure au temps 1 du sujet X_i_ et X_i2_ sa mesure au temps 2

On testera 3 effets en comparant les moyennes sur les variables *Y_i_, Z_i_* et *W_i_* entre les séquences S_1_ et S_2_:

- Un effet groupe ou séquence thérapeutique à partir de la variable *Y_i_* ainsi définie : $Y_{i}=X_{i1}+X_{i2}, \forall i\in\left\{S_{1} ou S_{2}\right\}$
- Un effet traitement, comparant directement l’effet de l’ingestion de glutamine au placebo à partir de la variable *Z_i_* ainsi définie : $Z_{i}=X_{i1}-X_{i2}, \forall i\in\left\{S_{1} ou S_{2}\right\}$
- Un effet ordre ou temps, comparant le placebo à l’ingestion de glutamine, à partir de la variable *W_i_* ainsi définie : $W_{i}=X_{i1}-X_{i2} si i\in S_{1}$ et $W_{j}=X_{j1}-X_{j2} si j\in S_{2}$

Les tests de comparaison de moyennes seront paramétriques (test t de Student) ou non paramétriques (test de Wilcoxon) selon la nature Gaussienne ou non des variables.

Cette même analyse sera effectuée sur les critères de jugement secondaires de l’essai.

Dans l’hypothèse de la sortie prématurée de l’étude d’un sujet, le sujet ne sera pas remplacé.

- **Responsable de l’analyse des données et logiciels de travail**

L’analyse statistique sera réalisée sous la responsabilité du Docteur Corinne Alberti, PH dans le service de Santé Publique de l’hôpital Robert Debré. Les données seront saisies par une société de service extérieure à l’hôpital et l’analyse statistique sera effectuée à l’aide du logiciel SAS v 8.2 (Cary system).

**Produit thérapeutique**

La **L-Glutamine** est un acide aminé. Sa biodisponibilité a été étudiée par voie entérale (7, 40), intraveineuse (54, 55), et sous forme de dipeptides pour perfusion intraveineuse. La glutamine sera préparée à partir d’un lot unique de qualité pharmaceutique conformément aux bonnes pratiques en pharmacie et en recherche clinique par le Secteur Essais Cliniques de l’AGEPS.

A ce jour, aucune étude n’a évalué le bénéfice fonctionnel de la prise de glutamine dans cette indication. La comparaison à un **placebo** (témoin de l’évolution spontanée de la maladie) est indispensable pour porter avec rigueur l’indication de cette supplémentation nutritionnelle. Il ne serait pas justifié de proposer la contrainte d’une supplémentation prolongée si aucun effet n’était observé. Le placebo sera préparé et conditionné dans le respect des bonnes pratiques pharmaceutiques. La présentation sera identique à la glutamine (masse et conditionnement).

Les préparations seront adaptées au poids de l’enfant à la visite d’inclusion et préparées pour la durée totale de l’étude. La **dose**(0.5g/kg/j) est identique à celle que nous avons précédemment utilisée (1-3, 7). La randomisation qui déterminera l’ordre dans lequel glutamine et placebo seront administrés sera gérée par la DRCD en collaboration avec l’AGEPS (Enveloppes de randomisation par blocs équilibrés de 6 patients).

**Durée de supplémentation.** Peu d’études ont observé un bénéfice fonctionnel dans la myopathie de Duchenne de Boulogne. La prise de corticoïdes est associée à une amélioration de la force musculaire, des paramètres de marche et à une augmentation de la créatininurie (52). L’effet sur la force musculaire croît jusqu’à 4 mois puis se stabilise. Le choix de 4 mois de traitement permettra de mettre en évidence un effet s’il existe en limitant la contrainte d’un traitement prolongé non répété.

La durée de la **fenêtre thérapeutique** est fixée empiriquement à 1 mois. Plusieurs études ont évalué la glutamine selon un schéma expérimental en cross over (37-39, 56-60), 2 études seulement incluaient une fenêtre thérapeutique pour des raisons indépendantes à la glutamine (37, 60). Le renouvellement plasmatique de la glutamine est rapide (350µmol/kg/h chez l’adulte et plus chez l’enfant)(7, 48). Le contenu corporel total de glutamine libre qui représente 60% des acides aminés libres de l’organisme (42) est totalement renouvelé en moins de 24h ce qui suggère une faible durée de rémanence pour un effet associé à la glutamine.

Chaque enfant recevra en une dispensation unique au début de chaque période les unités thérapeutiques adaptées à son poids (visite d’inclusion) et conditionnées dans des sachets identifiés.

L’**observance** sera évaluée sur les sachets vides rendus et la déclaration du patient et/ou de sa famille (carnet de traitement remis à la famille). La **tolérance clinique** du supplément sera évaluée par un questionnaire type à chaque visite (goût, nausées, vomissement, humeur du patient).

Evenements indésirables

Au cours du précédent essai nous n’avons observé aucun evenement indésirable grave (EIG) (3) (Etude de promotion AP-HP, terminée en 2002). De plus, aucun EIG n’a été observé dans la littérature (54, 55).

Un malaise vagal ou une veinite peuvent survenir pour toute prise de sang. Cependant, le risque de veinite est ici mineur car il n’y aura aucune perfusion de soluté. Nous avons observé une augmentation de l’urée sanguine au cours du précédent essai due à l’augmentation de l’apport en protéines(3) (Etude de promotion AP-HP, terminée en 2002). Par contre, la concentration restait dans les limites de la normale. Ces EI ne peuvent être considerés comme graves « stricto sensu ». Voir le site web: http:/ctep.info.nih.gov/reporting/index.html pour verifier les classifications et les niveaux d’evenements indésirables.

Il sera donné à l’enfant une carte précisant qu’il participe à une étude clinique sur laquelle seront portées les consignes à suivre en cas d’effets indésirable quel qu’il soit.En cas de nécessité médicale la levée d'insu pourra être demandée par un médecin au centre anti-poison de l'hôpital Fernand Widal de Paris (Tel : 01 40 25 48 48).

**Faisabilité**

Cette étude **tricentrique inter-CIC** sera réalisée dans les CIC du CHR&U de Lille et de l’hôpital Robert Debré à Paris et dans le service de Pédiatrie du CHU de Poitiers. Les deux CIC jouent un rôle clef dans la fiabilité du recueil de données et la rigueur du suivi du protocole. La création et la gestion du cahier d’observation seront réalisées par les Assistants de Recherche Clinique des deux CIC. Les fichiers informatiques de traitement des données seront déclarés à la Commission Nationale Informatique et Libertés (CNIL). L’étude bénéficie enfin de l’expertise logistique et de gestion de l’**URC Paris-Nord** (Pr Philippe RAVAUD) par son antenne à Robert Debré.

Le recrutement se fera à partir des consultations multidisciplinaires de la myopathie à Paris, Poitiers et Lille. Ces collaborations ont permis d’inclure 26 patients en moins de 6 mois dans un précédent essai (3).

L’information de cette étude sera réalisée par l’intermédiaire du journal associatif Vaincre La Myopathie (VLM).

La réalisation de l’étude se fera en accord avec les principes de l’éthique en Recherche Biomédicale (Déclaration d’Helsinki) et les termes de la loi Huriet (Dec 1988 révisée). Elle sera présentée à un Comite de Protection des Personnes se prêtant à une Recherche Biomédicale (CCPPRB) avec l’avis de promotion.

Les parents et les enfants seront informés oralement et par écrit et leur consentement signé sera demandé.

**Perspectives**

*Dans la myopathie de Duchenne de Boulogne*

Un effet objectif sur 4 mois incitera à proposer de la glutamine sur de plus longues durées. De même cette prise en charge nutritionnelle devra être évaluée chez l’enfant ayant déjà perdu la marche afin de mettre en évidence un bénéfice fonctionnel plus discret mais déterminant pour la vie de tous les jours de ces enfants (autonomie pour manger, tonus, …).

L’objectif est de freiner la progression de la maladie. Dans la MDB un effet clinique de la glutamine contribuera à mener les enfants à la thérapie génique dans les meilleures conditions.

*Dans les pathologies chroniques qui s’accompagnent d’un catabolisme musculaire*

L’enjeu est d’améliorer la prise en charge nutritionnelle dans les pathologies chroniques notamment en modulant le métabolisme par certains nutriments (Nutri-thérapie). Nous avons montré que la glutamine inhibe la dégradation protéique de façon spécifique (3). La mise en évidence d’un bénéfice fonctionnel dans la myopathie de Duchenne de Boulogne sera un argument fort pour étendre cette démarche d’évaluation chez l’enfant en milieu de réanimation, au cours du stress chirurgical, de la corticothérapie prolongée, …

**Tableau I : Calendrier des visites**

|  |  | **Période 1** | | | **Période 2** | | |
| --- | --- | --- | --- | --- | --- | --- | --- |
|  | **Inclusion** | **M0** | **M2** | **M4** | **M5** | **M7** | **M9** |
| **Critères d’Inclusion** | ☺ |  |  |  |  |  |  |
| **Examen Clinique** | ☺ | ☺ | ☺ | ☺ | ☺ | ☺ | ☺ |
| **Vitesse de marche** |  | ☺ | ☺ | ☺ | ☺ | ☺ | ☺ |
| **Tolérance/Observance** |  |  | ☺ | ☺ |  | ☺ | ☺ |
| **Biologie Sang** |  | ☺ | ☺ | ☺ | ☺ | ☺ | ☺ |
| **Biologie Urinaire** |  | ☺ | ☺ | ☺ | ☺ | ☺ | ☺ |
| **DXA** |  |  |  | ☺ |  |  | ☺ |
| **BIA** |  | ☺ | ☺ | ☺ | ☺ | ☺ | ☺ |
| **Diététique** |  |  |  | ☺ |  |  | ☺ |

**Légende** : L’allocation de glutamine ou placebo aux deux périodes se fera par tirage au sort; La **visite d'inclusion** aura lieu dans les 2 mois qui précède M0 (au minimum 7 jours avant M0, tolérance d’une semaine pour la programmation des visites ; **Biologie sang** : Ionogramme sanguin avec urée et créatinine, Enzymes hépatiques : SGOT, SGPT, CPK, P Alc., Hormones : Glycémie et insulinémie à jeun, IgFI, IgFBP1; **Biologie urinaire** : Créatininurie des 24h sur 3 jours, rapport 3-MH/créatinine  ; **BIA** : impédancemétrie monofréquence ; **DXA** : absorptiométrie biphotonique

Bibliographie

1. Hankard RG, Haymond MW, Darmaun D 1996 Effect of glutamine on leucine metabolism in humans. Am J Physiol 271:E748-754.

2. Hankard RG, Hammond D, Haymond MW, Darmaun D 1998 Oral glutamine slows down whole body protein breakdown in Duchenne muscular dystrophy. Pediatr Res 43:222-226.

3. Eléouet C, Rigal O, Fontan JE, Michel C, Levy-Marchal C, Hankard R 2002 Réponse du métabolisme protéique à 10 jours de supplémentation orale en glutamine chez l'enfant myopathe. Nutr Clin Metab 16:7-28.

4. Granchelli JA, Pollina C, Hudecki MS 2000 Pre-clinical screening of drugs using the mdx mouse. Neuromuscul Disord 10:235-239.

5. Haussinger D, Lang F, Gerok W 1994 Regulation of cell function by the cellular hydration state. Am J Physiol 267:E343-355.

6. Quillard M, Husson A, Lavoinne A 1996 Glutamine increases argininosuccinate synthetase mRNA levels in rat hepatocytes. The involvement of cell swelling. Eur J Biochem 236:56-59.

7. Hankard RG, Darmaun D, Sager BK, D'Amore D, Parsons WR, Haymond M 1995 Response of glutamine metabolism to exogenous glutamine in humans. Am J Physiol 269:E663-670.

8. Smith RJ, Larson S, Stred SE, Durschlag RP 1984 Regulation of glutamine synthetase and glutaminase activities in cultured skeletal muscle cells. J Cell Physiol 120:197-203.

9. Labow BI, Souba WW, Abcouwer SF 1999 Glutamine synthetase expression in muscle is regulated by transcriptional and posttranscriptional mechanisms. Am J Physiol 276:E1136-1145.

10. Coeffier M, Le Pessot F, Leplingard A, Marion R, Lerebours E, Ducrotte P, Dechelotte P 2002 Acute enteral glutamine infusion enhances heme oxygenase-1 expression in human duodenal mucosa. J Nutr 132:2570-2573.

11. Zhou X, Thompson JR 1997 Regulation of protein turnover by glutamine in heat-shocked skeletal myotubes. Biochim Biophys Acta 1357:234-242.

12. Bruhat A, Jousse C, Wang XZ, Ron D, Ferrara M, Fafournoux P 1997 Amino acid limitation induces expression of CHOP, a CCAAT/enhancer binding protein-related gene, at both transcriptional and post-transcriptional levels. J Biol Chem 272:17588-17593.

13. Abcouwer SF, Schwarz C, Meguid RA 1999 Glutamine deprivation induces the expression of GADD45 and GADD153 primarily by mRNA stabilization. J Biol Chem 274:28645-28651.

14. Fafournoux P, Bruhat A, Jousse C 2000 Amino acid regulation of gene expression. Biochem J 351:1-12.

15. Rhoads JM, Argenzio RA, Chen W, Rippe RA, Westwick JK, Cox AD, Berschneider HM, Brenner DA 1997 L-glutamine stimulates intestinal cell proliferation and activates mitogen-activated protein kinases. Am J Physiol 272:G943-953.

16. Haussinger D, Schliess F, Dombrowski F, Vom Dahl S 1999 Involvement of p38MAPK in the regulation of proteolysis by liver cell hydration. Gastroenterology 116:921-935.

17. Dennis PB, Fumagalli S, Thomas G 1999 Target of rapamycin (TOR): balancing the opposing forces of protein synthesis and degradation. Curr Opin Genet Dev 9:49-54.

18. Powell-Tuck J 1999 Total parenteral nutrition with glutamine dipeptide shortened hospital stays and improved immune status and nitrogen economy after major abdominal surgery. Gut 44:155.

19. Stehle P, Zander J, Mertes N, Albers S, Puchstein C, Lawin P, Furst P 1989 Effect of parenteral glutamine peptide supplements on muscle glutamine loss and nitrogen balance after major surgery. Lancet 1:231-233.

20. Morlion BJ, Stehle P, Wachtler P, Siedhoff HP, Koller M, Konig W, Furst P, Puchstein C 1998 Total parenteral nutrition with glutamine dipeptide after major abdominal surgery: a randomized, double-blind, controlled study. Ann Surg 227:302-308.

21. Griffiths RD, Jones C, Palmer TE 1997 Six-month outcome of critically ill patients given glutamine-supplemented parenteral nutrition. Nutrition 13:295-302.

22. Griffiths RD, Allen KD, Andrews FJ, Jones C 2002 Infection, multiple organ failure, and survival in the intensive care unit: influence of glutamine-supplemented parenteral nutrition on acquired infection. Nutrition 18:546-552.

23. Jones C, Palmer TE, Griffiths RD 1999 Randomized clinical outcome study of critically ill patients given glutamine-supplemented enteral nutrition. Nutrition 15:108-115.

24. Hammarqvist F, Wernerman J, von der Decken A, Vinnars E 1990 Alanyl-glutamine counteracts the depletion of free glutamine and the postoperative decline in protein synthesis in skeletal muscle. Ann Surg 212:637-644.

25. Hammarqvist F, Wernerman J, Ali R, von der Decken A, Vinnars E 1989 Addition of glutamine to total parenteral nutrition after elective abdominal surgery spares free glutamine in muscle, counteracts the fall in muscle protein synthesis, and improves nitrogen balance. Ann Surg 209:455-461.

26. Long CL, Nelson KM, DiRienzo DB, Weis JK, Stahl RD, Broussard TD, Theus WL, Clark JA, Pinson TW, Geiger JW, et al. 1995 Glutamine supplementation of enteral nutrition: impact on whole body protein kinetics and glucose metabolism in critically ill patients. JPEN J Parenter Enteral Nutr 19:470-476.

27. van Acker BA, Hulsewe KW, Wagenmakers AJ, von Meyenfeldt MF, Soeters PB 2000 Response of glutamine metabolism to glutamine-supplemented parenteral nutrition. Am J Clin Nutr 72:790-795.

28. Ziegler TR, Young LS, Benfell K, Scheltinga M, Hortos K, Bye R, Morrow FD, Jacobs DO, Smith RJ, Antin JH, et al. 1992 Clinical and metabolic efficacy of glutamine-supplemented parenteral nutrition after bone marrow transplantation. A randomized, double-blind, controlled study. Ann Intern Med 116:821-828.

29. MacBurney M, Young LS, Ziegler TR, Wilmore DW 1994 A cost-evaluation of glutamine-supplemented parenteral nutrition in adult bone marrow transplant patients. J Am Diet Assoc 94:1263-1266.

30. Dechelotte P, Bleichner G, Hasselmann M, Dassonville JM, Hecketsweiler B, Cynober L, Czernichow P, Rangaraj J 2002 La supplémentation en alanyl-glutamine (Dipeptiven) de la nutrition parentérale (NPT) réduit le taux de complications infectieuses chez les patients de réanimation. Etude multicentrique française. Nutr Clin Metab 16:7-28.

31. Houdijk AP, Rijnsburger ER, Jansen J, Wesdorp RI, Weiss JK, McCamish MA, Teerlink T, Meuwissen SG, Haarman HJ, Thijs LG, van Leeuwen PA 1998 Randomised trial of glutamine-enriched enteral nutrition on infectious morbidity in patients with multiple trauma. Lancet 352:772-776.

32. Darmaun D, Roig JC, Auestad N, Sager BK, Neu J 1997 Glutamine metabolism in very low birth weight infants. Pediatr Res 41:391-396.

33. Lacey JM, Crouch JB, Benfell K, Ringer SA, Wilmore CK, Maguire D, Wilmore DW 1996 The effects of glutamine-supplemented parenteral nutrition in premature infants. JPEN J Parenter Enteral Nutr 20:74-80.

34. des Robert C, Le Bacquer O, Piloquet H, Roze JC, Darmaun D 2002 Acute effects of intravenous glutamine supplementation on protein metabolism in very low birth weight infants: a stable isotope study. Pediatr Res 51:87-93.

35. Neu J, Roig JC, Meetze WH, Veerman M, Carter C, Millsaps M, Bowling D, Dallas MJ, Sleasman J, Knight T, Auestad N 1997 Enteral glutamine supplementation for very low birth weight infants decreases morbidity. J Pediatr 131:691-699.

36. Dallas MJ, Bowling D, Roig JC, Auestad N, Neu J 1998 Enteral glutamine supplementation for very-low-birth-weight infants decreases hospital costs. JPEN J Parenter Enteral Nutr 22:352-356.

37. Seguy D, Vahedi K, Kapel N, Souberbielle JC, Messing B 2003 Low-dose growth hormone in adult home parenteral nutrition-dependent short bowel syndrome patients: a positive study. Gastroenterology 124:293-302.

38. Jeppesen PB, Szkudlarek J, Hoy CE, Mortensen PB 2001 Effect of high-dose growth hormone and glutamine on body composition, urine creatinine excretion, fatty acid absorption, and essential fatty acids status in short bowel patients: a randomized, double-blind, crossover, placebo-controlled study. Scand J Gastroenterol 36:48-54.

39. Scolapio JS, Camilleri M, Fleming CR, Oenning LV, Burton DD, Sebo TJ, Batts KP, Kelly DG 1997 Effect of growth hormone, glutamine, and diet on adaptation in short-bowel syndrome: a randomized, controlled study. Gastroenterology 113:1074-1081.

40. Dechelotte P, Darmaun D, Rongier M, Hecketsweiler B, Rigal O, Desjeux JF 1991 Absorption and metabolic effects of enterally administered glutamine in humans. Am J Physiol 260:G677-682.

41. Darmaun D, Just B, Messing B, Rongier M, Thuillier F, Koziet J, Grasset E 1994 Glutamine metabolism in healthy adult men: response to enteral and intravenous feeding. Am J Clin Nutr 59:1395-1402.

42. Bergstrom J, Furst P, Noree LO, Vinnars E 1974 Intracellular free amino acid concentration in human muscle tissue. J Appl Physiol 36:693-697.

43. Hankard RG, Haymond MW, Darmaun D 1997 Role of glutamine as a glucose precursor in fasting humans. Diabetes 46:1535-1541.

44. Nurjhan N, Bucci A, Perriello G, Stumvoll M, Dailey G, Bier DM, Toft I, Jenssen TG, Gerich JE 1995 Glutamine: a major gluconeogenic precursor and vehicle for interorgan carbon transport in man. J Clin Invest 95:272-277.

45. Cersosimo E, Williams P, Hoxworth B, Lacy W, Abumrad N 1986 Glutamine blocks lipolysis and ketogenesis of fasting. Am J Physiol 250:E248-252.

46. Windmueller HG, Spaeth AE 1990 Uptake and metabolism of plasma glutamine by the small intestine. Nutr Rev 48:310-312.

47. Darmaun D, Messing B, Just B, Rongier M, Desjeux JF 1991 Glutamine metabolism after small intestinal resection in humans. Metabolism 40:42-44.

48. Hankard R, Goulet O, Ricour C, Rongier M, Colomb V, Darmaun D 1994 Glutamine metabolism in children with short-bowel syndrome: a stable isotope study. Pediatr Res 36:202-206.

49. Houtkooper LB, Lohman TG, Going SB, Hall MC 1989 Validity of bioelectric impedance for body composition assessment in children. J Appl Physiol 66:814-821.

50. Beghin L, Michel C, Fontan JE, Gottrand F, Cuisset JM, Hankard R 2002 Validité de la mesure de la composition corporelle par impédancemètrie mono-fréquence dans la myopathie. Nutr Clin Metab 16:29-45.

51. Zatz M, Rapaport D, Vainzof M, Passos-Bueno MR, Bortolini ER, Pavanello Rde C, Peres CA 1991 Serum creatine-kinase (CK) and pyruvate-kinase (PK) activities in Duchenne (DMD) as compared with Becker (BMD) muscular dystrophy. J Neurol Sci 102:190-196.

52. Mendell JR, Moxley RT, Griggs RC, Brooke MH, Fenichel GM, Miller JP, King W, Signore L, Pandya S, Florence J, et al. 1989 Randomized, double-blind six-month trial of prednisone in Duchenne's muscular dystrophy. N Engl J Med 320:1592-1597.

53. Bowen TR, Miller F, Mackenzie W 1999 Comparison of oxygen consumption measurements in children with cerebral palsy to children with muscular dystrophy. J Pediatr Orthop 19:133-136.

54. Ziegler TR, Benfell K, Smith RJ, Young LS, Brown E, Ferrari-Baliviera E, Lowe DK, Wilmore DW 1990 Safety and metabolic effects of L-glutamine administration in humans. JPEN J Parenter Enteral Nutr 14:137S-146S.

55. Lowe DK, Benfell K, Smith RJ, Jacobs DO, Murawski B, Ziegler TR, Wilmore DW 1990 Safety of glutamine-enriched parenteral nutrient solutions in humans. Am J Clin Nutr 52:1101-1106.

56. Anderson PM, Schroeder G, Skubitz KM 1998 Oral glutamine reduces the duration and severity of stomatitis after cytotoxic cancer chemotherapy. Cancer 83:1433-1439.

57. Antonio J, Sanders MS, Kalman D, Woodgate D, Street C 2002 The effects of high-dose glutamine ingestion on weightlifting performance. J Strength Cond Res 16:157-160.

58. Krzywkowski K, Petersen EW, Ostrowski K, Kristensen JH, Boza J, Pedersen BK 2001 Effect of glutamine supplementation on exercise-induced changes in lymphocyte function. Am J Physiol Cell Physiol 281:C1259-1265.

59. Scolapio JS, McGreevy K, Tennyson GS, Burnett OL 2001 Effect of glutamine in short-bowel syndrome. Clin Nutr 20:319-323.

60. Szkudlarek J, Jeppesen PB, Mortensen PB 2000 Effect of high dose growth hormone with glutamine and no change in diet on intestinal absorption in short bowel patients: a randomised, double blind, crossover, placebo controlled study. Gut 47:199-205.
