# Supplementary material for: Lack of Functional Benefit with Glutamine versus Placebo in Duchenne Muscular Dystrophy: A Randomized Crossover Trial
Source: PLoS One. 2009 May 6;4(5):e5448. doi: 10.1371/journal.pone.0005448 (PMC2673684; doi:10.1371/journal.pone.0005448)
Supplement: Statement S1 — Statement from the ethics committee indicating their approval of the research (in French) (1.19 MB PDF) [file pone.0005448.s004.pdf]

COMITE CONSULTATIF DE PROTECTION DES PERSONNES DANS LA RECHERCHE BIOMEDICALE DE PARIS BICHAT-CLAUDE BERNARD

(AGREMENT DU 23 JUILLET 1991. JO DU 02 AOUT 1991)

CCPPRB

DR RENAUD GRUAT

CENTRE HOSPITALIER RENE DUBOS

6, AVENUE DE L'ILE DE FRANCE

BOITE POSTALE 79 PONTOISE

95303 CERGY-PONTOISE

TEL : 01-30-75-41-76

FAX : 01-30-75-40-13

Pontoise, le 20 septembre 2004

Dr régis Hankard
Centre d'investigation clinique
Hôpital Robert Debré
48, bd Sérurier
75019 Paris

Membres du Comité présents à la séance du 15 septembre 2004

Président
R Gruat

Secrétaire de séance
R Gruat

Personnes qualifiées en matière de recherche biomédicale
D.Lebrec
R.Farinotti

Personnes qualifiées en matière de psychologie
P.Nominé

Personnes qualifiées en matière sociale
M.Pinet-Ribeiro

Délibération

Projet enregistré sous le n ° : 2004/13

Le Comité a été saisi le par le Docteur régis Hankard d'une demande d'avis, concernant un projet ***Avec Bénéfice Individuel direct***, (enregistré sous le n° 2004/13) intitulé

AOM 03121 EFFET DE LA PRISE ORALE DE GLUTAMINE SUR LA FONCTION ET LA MASSE MUSCULAIRE DANS LA MYOPATHIE DE DUCHENNE DE BOULOGNE

Dont le promoteur est l'Aphp

Après avoir étudié les réponses apportées et en avoir délibéré, le Comité décide de lever la réserve du 07 juillet 2004 et d'adopter l'avis suivant:

Avis Favorable

Le Président
