# Supplementary material for: Lack of Functional Benefit with Glutamine versus Placebo in Duchenne Muscular Dystrophy: A Randomized Crossover Trial
Source: PLoS One. 2009 May 6;4(5):e5448. doi: 10.1371/journal.pone.0005448 (PMC2673684; doi:10.1371/journal.pone.0005448)
Supplement: Form S1 — Sample of a patient (parent) consent form (in French) (0.11 MB PDF) [file pone.0005448.s005.pdf]

Formulaire d'information et de consentement pour une personne participant à une recherche biomédicale

Effet de la prise orale de glutamine sur la fonction et la masse musculaire dans la myopathie de Duchenne de Boulogne

Les pages de ce document doivent être numérotées (1/nombre total de pages), paraphées par le médecin investigateur et les personnes donnant leur consentement ; la dernière page doit être datée et signée par ces mêmes personnes.

Le docteur(1)………………..….., médecin investigateur nous a proposé que notre enfant (Nom, Prénom) .... participe à la recherche biomédicale intitulée "***Effet de la prise orale de glutamine sur la fonction et la masse musculaire dans la myopathie de Duchenne de Boulogne***".

L'Assistance Publique-Hôpitaux de Paris, promoteur de cet essai, a contracté une assurance conformément à la loi. Le médecin nous a précisé que nous étions libres d'accepter ou de refuser qu'il participe à cette recherche.

Afin d'éclairer notre décision, nous avons reçu et bien compris les informations suivantes :

La **glutamine**, est présente dans les protéines que nous mangeons tous les jours (viande, poisson, œufs). Elle freine la destruction des protéines du corps lorsqu'elle est donnée en plus de l'alimentation normale d'enfants atteints de myopathie de Duchenne de Boulogne pendant 10 jours.

L'étude à laquelle nous souhaitons que votre enfant participe teste si la glutamine est capable de réduire la destruction des protéines du muscle et donc de limiter le handicap lié à la maladie. Nous avons calculé que 30 enfants devaient participer pour mettre en évidence une amélioration minimale de 10%.

La quantité de glutamine est identique à celle que nous avons utilisé précédemment et dépend du poids de votre enfant à la première visite (0.5 g/kg/j). Cette quantité restera identique tout au long de l'étude et devra être prise par la bouche tous les jours.

L'étude dure 9 mois. Afin de bien tester l'effet de la glutamine sur l'évolution de la maladie, votre enfant recevra dans un ordre tiré au hasard, de la glutamine pendant 4 mois puis après une période de 1 mois pendant laquelle il ne prendra rien, un produit sans effet pendant 4 autres mois ou l'inverse. Ni vous, ni les médecins qui s'occupent de cette étude ne sauront ce que votre enfant prendra jusqu'à la fin de l'étude. Les deux poudres ont le même aspect et un même goût. C'est ce que l'on appelle une « **étude en double aveugle** ».

paraphe de l'investigateur :-------

paraphe des parents/titulaires de l'autorité parentale :-------

paraphe de l'enfant :-------

Il sera nécessaire de venir 6 fois à l'hôpital pendant une demi journée en plus de la première visite qui sert à expliquer l'étude et à vérifier que votre enfant peut y participer. Toutes ces visites se feront soit au centre d'investigation clinique de l'hôpital Robert Debré à Paris, soit au centre d'investigation clinique du CHR&U de Lille, soit enfin dans le service de Pédiatrie du CHU de Poitiers.

A chaque visite il sera pratiqué :

1) un examen clinique incluant un test où l'on demandera à votre enfant de marcher à son rythme sur une distance de 170m et sur le plat,

2) un interrogatoire,

3) une prise de sang (15 mL, l'équivalent d'une cuillère à soupe) qui sera réalisée 1h30 après la pose d'une crème qui atténue la douleur de la piqûre et qui permettra de voir comment fonctionnent le foie, les reins,… (2 mL seront conservés pour pouvoir réaliser des analyses qui seront demandées en fonction des résultats que nous obtiendrons),

4) des examens d'urines qui servent à estimer le poids des muscles du corps et leur destruction,

5) et une estimation de la quantité de graisses du corps à l'aide d'un appareil électrique et parfaitement indolore qui nécessite de coller des autocollants sur une main et un pied. Ce dernier examen sera complété par une mesure plus précise à la fin de chaque période de 4 mois qui fonctionne comme une « radio » mais nécessite 10 fois moins de rayons X (absorptiométrie biphotonique). Il nécessite de coucher l'enfant sur une table pendant une dizaine de minutes.

Cette étude est considérée « **avec bénéfice individuel direct** » car elle renforce le suivi de votre enfant et peut avoir des conséquences bénéfiques sur la maladie. Les contraintes de cette étude sont de prendre tous les jours, par la bouche, pendant deux périodes de 4 mois, un sachet de 20-30 g de poudre (5-6 cuillères à soupe remplies), de venir 7 fois à l'hôpital pendant une demi journée à des périodes définies. On vous demandera de ramener tous les sachets vides et de noter tout ce que votre enfant mange 1 jour de semaine et un jour de week-end dans la semaine qui précède la visite pour l'étude.

La glutamine est présente dans les aliments que nous mangeons tous les jours. La quantité que nous donnons correspond environ à un quart de steak de 100g. Les sachets de glutamine sont préparés par la Pharmacie Centrale des Hôpitaux de Paris avec la même rigueur que pour un médicament. Nous n'attendons aucun effet indésirable, néanmoins nous vous demanderons de bien vouloir noter sur un carnet tout signe que pourrait présenter votre enfant (nausée, vomissement, mal de tête, douleur de ventre, diarrhée, constipation, grattage, rougeur sur la peau, tristesse ou au

paraphe de l'investigateur :-------

paraphe des parents/titulaires de l'autorité parentale :-------

paraphe de l'enfant :-------

contraire excitation,…) mais aussi toute consultation, hospitalisation. Il vous sera donné une carte indiquant que vous faites partie de cet essai avec un numéro de téléphone que vous ou le médecin qui s'occupera de votre enfant pourra joindre à tout moment. Les examens qui seront réalisés sur votre enfant ne présentent aucun risque. Comme toute prise sang il faut savoir qu'une rougeur peut survenir après et il faudra nous en informer si cela se produisait.

Si la glutamine est capable d'améliorer la marche ou le poids des muscles après 4 mois elle pourrait contribuer à ralentir la progression de la maladie et il faudrait en donner très tôt et sur de longues périodes ce qui serait l'objet de nouvelles études. Il faudrait sûrement aussi la tester dans d'autres maladies qui s'accompagnent d'une perte de muscle.

Cette recherche a reçu l'avis favorable du Comité Consultatif de Protection des Personnes participant à une Recherche Biomédicale de Paris Bichat Claude Bernard le 07/07/2004. <u>Aucune rémunération ne sera versée pour participer à cette recherche</u>. Par contre tous les frais (hospitalisation, examens, etc…) sont couverts par le projet de recherche qui a obtenu un financement national.

Le fichier informatique utilisé pour la recherche a fait l'objet d'une autorisation auprès de la Commission Nationale de l'Informatique et des Libertés en application des articles 40-1 et suivants de la loi " informatique et libertés. Nous acceptons que les données médicales le concernant ainsi que celles relatives à ses habitudes de vie recueillies à l'occasion de cette recherche puissent faire l'objet d'un traitement informatisé par les organisateurs de la recherche. Le droit d'accès et de rectification prévu par la loi " Informatique et Liberté " s'exerce à tout moment auprès des responsables de l'étude.

Pour toutes les informations de nature médicale, nous exercerons ce droit directement ou par l'intermédiaire d'un médecin de notre choix, le Dr……………………..(article 40 de la loi 78.17 du 6 janvier 1978 et art. L.1111-7 du Code de la Santé Publique). Les données recueillies demeureront strictement confidentielles. Elles ne pourront être consultées que par l'équipe médicale, les personnes dûment mandatées par le promoteur de la recherche et éventuellement par des représentants des autorités sanitaires et judiciaires habilitées.

Après en avoir discuté et avoir obtenu réponse à toutes nos questions, nous acceptons librement et volontairement que notre enfant participe à la recherche décrite ci-dessus. Nous sommes parfaitement conscients que nous pouvons retirer à tout moment notre consentement à sa participation à cette recherche et cela quelles que soient nos raisons et sans supporter aucune responsabilité. Le fait de ne plus participer à cette recherche ne portera pas atteinte à nos relations avec le médecin investigateur qui nous proposera, si nous le souhaitons et si besoin, une autre prise en charge pour notre enfant.

paraphe de l'investigateur :-------

paraphe des parents/titulaires de l'autorité parentale :-------

paraphe de l'enfant :-------

Nous pourrons à tout moment demander toute information complémentaire au Dr………………. (n° de téléphone).

Si nous le souhaitons, à son terme, nous serons informé(es) par l'investigateur qui recueille notre consentement des résultats globaux de cette recherche.

Notre consentement ne décharge en rien l'investigateur et le promoteur de l'ensemble de leurs responsabilités et nous et notre enfant conservons tous nos droits garantis par la loi.

L'investigateur :

Nom, prénom :
Fait à , le :
Signature

Signature des titulaires de l'exercice de l'autorité parentale :

Nom, prénom :

Fait à , le :

Signature

Nom, prénom :

Fait à , le :

Signature

NB : si l'un des parents est seul détenteur de l'autorité parentale, porter la mention : »je détiens seul l'autorité parentale «

Le consentement de l'enfant doit également être recherché s'il est apte à exprimer sa volonté. Il ne peut être passé outre à son refus ou à la révocation de son consentement.

L'enfant :

Nom, prénom :

Fait à , le :

Signature

**Ce document est à réaliser en 3 exemplaires originaux, dont l'un doit être gardé 15 ans par l'investigateur, un autre remis à la personne donnant son consentement et le troisième transmis au promoteur.**
