# Supplementary material for: Lack of Functional Benefit with Glutamine versus Placebo in Duchenne Muscular Dystrophy: A Randomized Crossover Trial
Source: PLoS One. 2009 May 6;4(5):e5448. doi: 10.1371/journal.pone.0005448 (PMC2673684; doi:10.1371/journal.pone.0005448)
Supplement: Form S2 — Sample of a patient (children) assent form (in French) (0.06 MB PDF) [file pone.0005448.s006.pdf]

Formulaire d'information destiné à un enfant participant à la recherche biomédicale :

« Effet de la prise orale de glutamine sur la fonction et la masse musculaire dans la myopathie de Duchenne de Boulogne »

La glutamine est un « acide aminé » c'est-à-dire une partie des protéines (viande, poisson, œuf) que l'on mange tous les jours.

Nous avons montré que la **glutamine** pouvait donner de la force chez des enfants atteints d'une maladie qui fait manquer de muscles : la myopathie de Duchenne de Boulogne.

Nous te proposons de participer à cette recherche pour voir si la glutamine permet d'améliorer le poids des muscles et la marche. Trente enfants comme toi vont participer à cette étude à Paris, à Lille et à Poitiers.

Tu peux accepter ou refuser d'y participer à tout moment, et nous respecterons ton choix. Ni toi, ni tes parents ne recevront de l'argent pour ta participation à cette étude, mais tout ce que nous ferons et le traitement seront gratuits.

Pour cela nous te demandons de manger tous les jours un sachet de poudre que tu peux mélanger avec ce que tu manges pendant 4 mois. Après un mois sans rien on te demandera de manger tous les jours un autre sachet de poudre pendant 4 autres mois. Cela dure donc 9 mois en tout. Une fois tu recevras de la glutamine et une autre fois une autre poudre mais ni toi, ni le médecin qui te suit ne sauront ce que tu prends jusqu'à ce qu'on ait fini l'étude.

Tu devras venir 6 fois à l'hôpital en plus de la première visite et à chaque fois on t'examinera, on te demandera de marcher, on te fera une prise de sang après t'avoir mis de la crème pour que ça fasse moins mal, on te demandera de faire pipi, on te collera des autocollants sur la peau pour savoir si tu as grossi et 2 fois on te mettra dans une machine qui voit aussi si tu as grossi.

Il faudra que tu nous rapportes les sachets vides, que tu notes avec tes parents ce que tu manges pendant 2 jours avant chaque visite et que tu nous dises si quelque chose ne va pas, si tu vas chez le docteur ou à l'hôpital ou encore si ton bras te fait mal ou est rouge après les prises de sang.

On te dira si ça marche ou pas. Si ce que l'on te donne marche, on pourra essayer d'en donner dès que l'on sait qu'un enfant a ta maladie ou sur de plus longues durées pour qu'il garde de la force le plus longtemps possible.
